# Potential Relationship between the Changes in Circulating microRNAs and the Improvement in Glycaemic Control Induced by Grape Pomace Supplementation

**DOI:** 10.3390/foods10092059

**Published:** 2021-09-01

**Authors:** Asier Léniz, Daniel Martínez-Maqueda, Alfredo Fernández-Quintela, Jara Pérez-Jiménez, María P. Portillo

**Affiliations:** 1 Araba Integrated Health Care Organization, Basque Health Service (Osakidetza), 01006 Vitoria-Gasteiz, Spain; asier.leniz@gmail.com; 2 Bioaraba Health Research Institute, 01006 Vitoria-Gasteiz, Spain; mariapuy.portillo@ehu.eus; 3 CIBER Fisiopatología de la Obesidad y Nutrición (CIBEROBN), Instituto de Salud Carlos III (ISCIII), 28222 Madrid, Spain; 4 Nutrition and Obesity Group, Department of Pharmacy and Food Sciences, Faculty of Pharmacy and Lucio Lascaray Research Center, University of the Basque Country (UPV/EHU), 01008 Vitoria-Gasteiz, Spain; 5 Department of Metabolism and Nutrition, Institute of Food Science, Technology and Nutrition (ICTAN-CSIC), José Antonio Novais 10, 28040 Madrid, Spain; d.martinez@outlook.es (D.M.-M.); jara.perez@ictan.csic.es (J.P.-J.); 6 Department of Agrifood Research, Madrid Institute for Rural, Agricultural and Food Research and Development (IMIDRA), A-2 Km. 38.2, 28805 Alcalá de Henares, Spain

**Keywords:** grape pomace, insulin response, microbiota, miR-30c, miR-122

## Abstract

MicroRNAs (miRNAs) represent important tools in medicine and nutrition as new biomarkers, and can act as mediators of nutritional and pharmacological interventions. The aim of the present study was to analyse the effect of grape pomace supplementation on the expression of seven selected miRNAs and their potential relationship with the observed positive effect on glycaemic control, in order to shed light on the mechanism underlying the beneficial effect of this dietary intervention. For this purpose, plasma samples were obtained from 49 subjects with metabolic syndrome. After supplementation with grape pomace (6 weeks), these subjects were categorised as responders (n = 23) or non-responders (n = 26) according to the changes in their fasting insulin rate. MiRNA expression at baseline and at the end of the supplementation was analysed by RT-PCR, and the MiRecords Database was used to identify potential target genes for the studied miRNAs. The increase observed in miR-23a in the whole cohort was present in both subgroups of participants. The increase in miR-181a was significant among non-responders but not responders. The decrease in miR-30c and miR-222 was found in the responders, but not in the non-responders. No changes were observed in miR-10a, miR-151a, miR-181a, and miR-let-7a expressions. After analysing these results, a potential involvement of the reduced expression of miR-30c and miR-222, two microRNAs associated with insulin resistance and diabetes, in the improvement of glycaemic control produced by grape pomace administration, can be proposed. Further research is needed to confirm the involvement of glycolytic enzymes, PI3K, AMPK, and IRS-1 in the effect of grape pomace, as suggested by the changes induced in microRNAs.

## 1. Introduction

MicroRNAs (miRNAs) are defined as small RNAs, with a short chain of nucleotides (18–22 units). Contrarily to mRNA, which encode for proteins in the body, miRNAs do not have this function [1]. They regulate the expression of genes encoding target proteins by binding to complementary target sequences in messenger RNA (mRNA), thus interfering with the protein translation process, or by mRNA destabilisation and degradation [2]. It has been estimated that almost 30% of our gene set is regulated by miRNAs. More than 1900 precursors and 2600 mature miRNAs have been identified in humans and the number continues to increase [3]. The miRNAs regulation network is rather complex because one miRNA can control the expression of various mRNA targets, whereas a single mRNA can be regulated by several miRNAs [3].

MiRNAs show a tissue-specific distribution, reporting different miRNA expressions depending on the analysed organ [4]. However, obtaining tissue biopsies in humans aimed at studying the miRNA profile can be a difficult, often traumatic, and non-practical process. In this scenario, circulating miRNAs represent an interesting option because they can be easily detected in serum and plasma in a non-invasive manner. They are transported from cell to cell either through gap junctions or through blood secretions, and they exert their effects on recipient cells. In blood, miRNAs are found in microvesicles, exosomes, and high-density lipoproteins, or are associated with RNA binding proteins [5]. Such circulating miRNAs are resistant to RNase activity, extreme pH, and temperature.

A large number of studies has demonstrated the relevant role of miRNAs in numerous biological functions, such as developmental timings, cell differentiation, embryogenesis, metabolism, organogenesis, and apoptosis [6]. It has also been shown that the circulating miRNAs vary under different physiological conditions, such as physical activity or pregnancy, and different pathological conditions, such as cancer, cardiovascular diseases, metabolic diseases, diabetes, and viral pathogenesis [7,8,9]. Thus, Kim et al. compared the expression of several miRNAs in visceral adipose tissue and blood from obese and non-obese subjects, and observed that the existing differences in visceral fat between obese and non-obese subjects were also present in their blood [10]. Consequently, miRNAs can play a role as diagnostic and prognostic biomarkers for several pathologies [11]. For example, circulating miR-208a can be a useful biomarker for the detection of an early myocardial injury in acute myocardial infarction in humans [12]. In a similar manner, circulating miR-122 may be regarded as a potential biomarker in monitoring the progression of histological changes in the liver and, for instance, in the evolution of liver steatosis to steatohepatitis [13].

Considering these alterations in miRNA profile observed in some pathologies, small non-coding mRNAs can represent potential innovative treatments against these diseases [14]. Indeed, miRNA-mediated therapies for treatment of cancer and chronic hepatitis C virus (HCV) infection have shown promising results in human Phase I clinical trials [15,16]. Moreover, miRNAs can also represent good biomarkers of response to different types of treatments, and thus differences in the circulating miRNA pattern can be useful to distinguish between responder and non-responder subjects. Hess et al. (2020) demonstrated that circulating levels of miR-222-3p significantly increased after an intervention consisting of caloric restriction treatment in obese patients, whereas miR-122-5p and miR-193a-5p were reduced [17].

Finally, miRNAs have been identified as mediators of the effects induced by a variety of drugs and compounds found in diets, such as phenolic compounds [18]. For instance, by studying the effects of proanthocyanidins on non-alcoholic fatty liver disease, Baselga-Escudero et al. (2014) showed that mir-122-5p mediated the effect of these compounds. Furthermore, the prevention of this liver alteration induced by resveratrol was reported to be mediated by mir-107-5p [19,20].

Taking the above into account, the aim of the present study was to analyse the potential involvement of miR-30c, miR-23a, miR-222, miR-let-7a, miR-151a, miR-181a, and miR-10a in the mechanisms of action underlying the positive effect of grape pomace supplementation, for 6 weeks, on glycaemic control in subjects exhibiting at least two factors for metabolic syndrome diagnosis.

## 2. Materials and Methods

### 2.1. Study Subjects and Experimental Design

This study was a randomised cross-over controlled clinical trial, carried out with permission obtained from the Ethics Subcommittee of the Spanish National Research Council (CSIC), Madrid, Spain (13 December 2016), and the Ethics Committee for Clinical Research of the University Hospital Puerta de Hierro-Majadahonda, Majadahonda, Spain (2 December 2016). Written informed consent was obtained from all subjects before their enrolment in the study, which was registered in the Clinical Trials database (NCT03076463).

**Figure 1:**
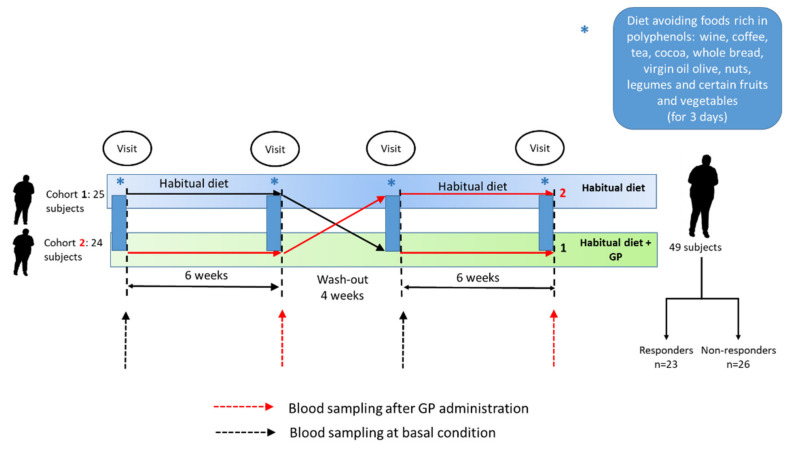
Experimental design of the randomised cross-over clinical trial. GP: grape pomace.

Subject recruitment was carried out using poster advertisements and mailing sent to different institutions. Inclusion criteria were age 18–70 years, in good health and fulfilling at least two of the following requirements for the diagnosis of MetS: body mass index >25 kg m^−2^; fasting glucose ≥100 mg dL^−1^; HDL-cholesterol ≤50 mg dL^−1^ in women and 40 mg dL^−1^ in men; triglycerides ≥150 mg dL^−1^; systolic pressure ≥130 mmHg or diastolic pressure ≥85 mmHg. Exclusion criteria were showing cardiometabolic diseases or pharmacological treatment for them, pregnancy or lactation, and current or close participation in any other dietary intervention study. After the recruitment period, 49 subjects with at least two factors of metabolic syndrome participated in the study and were randomly assigned either to the supplementation with grape pomace suspended in water (GP) or to the control (C) arm. No valid placebo was found for this period and, therefore, they followed their usual diet and had the same control as in the GP period. Both experimental periods had a duration of 6 weeks, separated by a 4 week wash-out. The GP for the six week trial was supplied to the participants at the beginning of the GP-supplementation period, who were required to keep it frozen. Two visits were scheduled at the beginning and at the end of each experimental period. Several cardiometabolic markers were assessed in biological samples at the beginning and at the end of the control period. No significant modifications were found, so the evaluation of miRNA was focused on samples at the beginning and at the end of the supplementation period (Figure 1). To avoid the potential effect of a high dietary consumption of phenolic compounds during the days previous to the visits, the subjects were required to discard from their diet foods rich in these compounds, such as wine, coffee, tea, cocoa, whole bread, virgin oil olive, nuts, legumes, and certain fruits and vegetables, such as berries or artichokes, 72 h prior to each visit day. For this purpose, volunteers were provided with a detailed list of foods to be avoided. At the end of the experimental period, 23 participants were classified as responder subjects and 26 as non-responders, based on a decrease in fasting insulin ≥10% [21]. Although the level of glucose remained unchanged, a strong reduction in insulin level was found in responder subjects (−40%) and, as a consequence, HOMA-IR was also significantly reduced [22].

GP was obtained from *Vitis vinifera* L., cv Tempranillo freshly collected at the moment of wine devatting (Roquesan Wineries, Quemada, Burgos, Spain). After transport at −20 °C, it was freeze dried, ground (particle size: 0.5 mm), sealed in monodoses (8 g), and kept at −20 °C. The content of extractable and non-extractable phenolic compounds, mostly as high molecular weight of proanthocyanidins in GP, was 6.2% and 23.4%, respectively. The methodology is described elsewhere [23]. Furthermore, a significant quantity of insoluble fibre (68.2%) was also determined by the indigestible fraction method [24].

### 2.2. Sample Collection and Serum Parameters

Blood samples were collected at the beginning of each period in fasting (overnight) conditions in BD Vacutainer venous collection tubes (cat #67525, Eysins, Vaud. Switzerland). Plasma was obtained by centrifugation (1000× *g*; 15 min). Plasma insulin was determined using commercial ELISA kits according to the manufacturer’s instructions (Merck-Millipore, Burlington, MS, USA). Fasting and postprandial blood glucose was measured by applying the enzyme electrode method using a Free Style Optimum Neo blood glucose meter from Abbott (Chicago, IL, USA).

### 2.3. Isolation and Quantitative Real-Time PCR of miRNAs

The miRNAs that were differentially expressed between responders and non-responders in a previous NGS analysis (miR-30c, miR-23a, miR-222, miR-let7a, miR-151a, miR-181a, and miR-10a) were studied in the present work [21]. The expression of these circulating mRNAs both at the beginning and at the end of the experimental period was analysed.

MiRNA extraction was carried out using a MiRNeasy Serum/Plasma Advanced Kit (Qiagen, Venlo, Limburg, The Netherlands) according to the manufacturer’s instructions. Total RNA containing miRNAs was diluted with RNase-free water (up to 20 μL). MiRNAs (2 μL of the RNA solution) were reverse-transcribed using the TaqMan^®^ Advanced miRNA cDNA Synthesis kit (Applied Biosystems, Foster City, CA, USA). Commercially available TaqMan probes (Thermo Fisher Scientific, Waltham, MA, USA) were used to determine miRNA concentrations.

A CFX96 Real-Time System (BioRad, Hercules, CA, USA) was used to detect and quantify individual miRNAs by RT-PCR. The reaction was set as follows: 95 °C for 20 s, 40 cycles of 95 °C for 3 s, and 60 °C for 30 s. All reactions were run in duplicate. The mRNA levels were normalised to the values of miRNA-191 (miR-191-5p: sequence 5′-CAACGGAAUCCCAAAAGCAGCUG-3′), and the results were expressed as fold changes of threshold cycle (Ct) value relative to the non-supplemented situation (basal situation) using the 2^−(ΔΔCt)^ method [25]. The sequences of targeted miRNA (source miRbase.org, accessed on 18 January 2020) are included in Supplemental Table S1.

### 2.4. Search for miRNAs Regulating Target Genes

The MiRecords database was used to identify validated and predicted target genes. Additionally, the Kyoto Encyclopaedia of Genes and Genomes (KEGG) database was utilised to identify the involvement of the predicted or validated target genes in metabolic pathways related to glycaemic control. Furthermore, a search of the literature was also performed.

### 2.5. Statistical Analysis

The Shapiro–Wilk test and Levene’s test were used to test normality and homoscedasticity of variance, respectively. Comparisons between two paired samples were drawn using a paired Student’s *t*-test or Wilcoxon’s signed rank test, as appropriate. All results are provided as mean values and standard errors. The Pearson’s or Spearman rho correlation coefficients were used for correlation analysis between miRNAs and levels of fasting glucose and insulin. In all cases, a *p*-value < 0.05 was considered as significant. IBM SPSS Statistics 24.0 (Armonk, NY, USA) was used to perform statistical analysis.

## 3. Results

**Figure 2:**
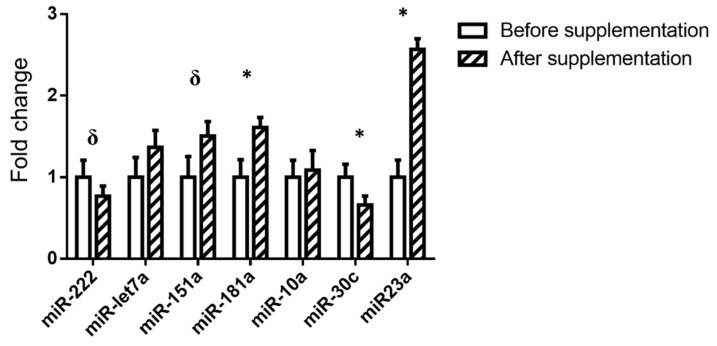
Relative miRNA expression in the whole cohort before and after the supplementation with grape pomace. The 2^-(ΔΔCt) method was used to calculate fold changes, relative to the expression before supplementation. Values are presented as mean ± standard error of the mean. Comparisons were carried out using paired Student’s *t*-test or Wilcoxon’s signed rank test when required. * *p* < 0.05, δ *p* < 0.1.

The expression of the miRNAs selected (miR-222, MiR-let-7a, miR-151a, miR-181a, miR-10a, miR-30c, and miR-23a) is shown in Figure 2. In the whole cohort, a significant increase was observed in miR-181 and miR-23a, and a significant decrease in miR-30c was revealed, after grape pomace supplementation. Moreover, a tendency towards a lower value emerged in the expression of miR-222 (−9.76%) and towards a higher value in miR-151a (+37.14%)

**Figure 3:**
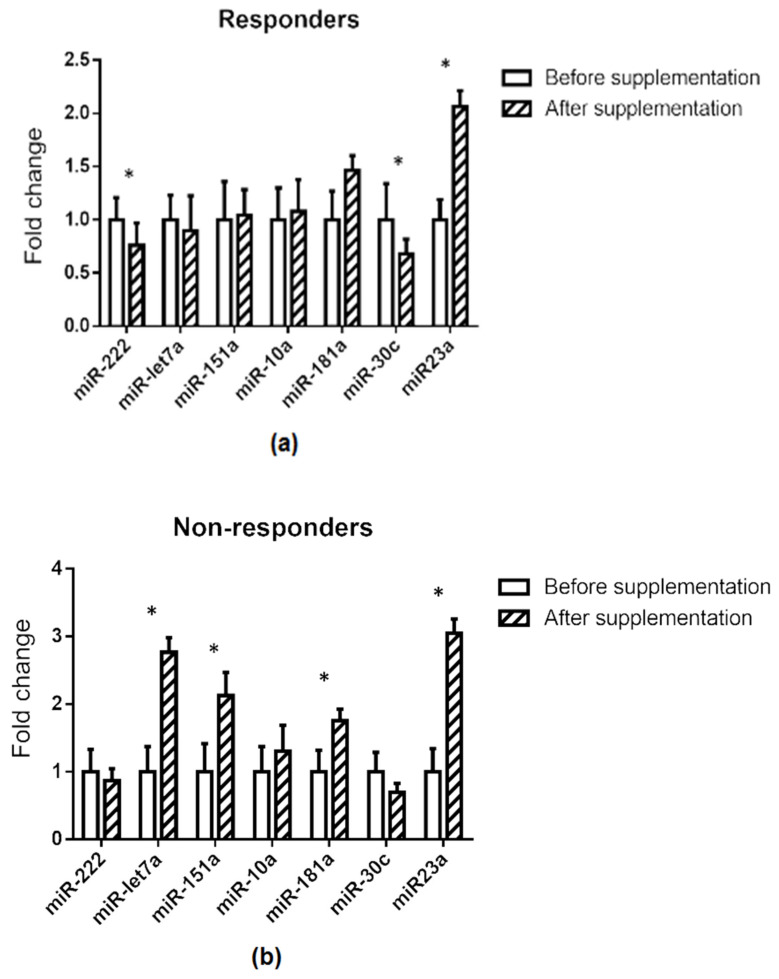
Relative miRNA expression before and after supplementation stratified in responders (**a**) and non-responders (**b**), based on fasting insulin reduction after supplementation. The 2^−(ΔΔCt)^ method was used to calculate fold changes relative to the expression before supplementation. Values are presented as mean ± standard error of the mean. Comparisons were carried out using the paired Student’s *t*-test or Wilcoxon’s signed rank test when required. * *p* < 0.05.

Differences in the pattern of response were observed when responders and non-responders were compared. Thus, whereas miR-222 and miR-30c showed significantly lower expression after grape pomace supplementation, this effect was not observed in the non-responders. By contrast, in the non-responders, miR-let7a, miR-181a, and miR-151a displayed significantly higher values after the supplementation with the grape pomace, but these miRNAs remained unchanged in the responders (Figure 3b). Finally, in both groups, miR-23a was significantly increased after grape pomace administration (Figure 3a,b).

The search for target genes related to glycaemic control in the MiRecords database did not yield any validated target gene. The predicted target genes are presented in Table 1. In addition, the search for target genes in the literature showed that IRS-1 and GLUT1 were direct targets for miR-122 and miR-10a, respectively.

In the present paper, the data concerning serum glucose and insulin are not presented because they were previously reported in other study. However, a correlation analysis was carried out using these data, revealing negative correlations between miR-222 and glucose (r = −0.335; *p* = 0.038), miR-222 and insulin (r = −0.310; *p* = 0.050), and miR-30a and insulin (r = −0.35; *p* = 0.044) [28].

## 4. Discussion

Circulating miRNAs have been widely studied as potential biomarkers in diverse pathologies, including those related to glucose homeostasis, due to their abundance and stability in several body fluids, including blood. In a previous study in which this cohort was examined, Next Generation Sequencing (NGS) analysis of miRNA was performed to identify biomarkers of response to grape pomace supplementation [29,30]. It was observed that subjects exhibiting a beneficial effect on glycaemic control after grape pomace administration (responders) were those showing a more impaired glycaemic control and, thus, with greater space for an improvement in insulin homeostasis. These subjects displayed an increased expression of seven microRNAs compared with those who did not respond [21]. In the present study, the aim was to analyse the effect of this dietary supplementation on the expression of seven selected miRNAs and their potential relationship with the observed positive effect on glycaemic control, in order to shed light on the mechanism underlying the beneficial effect of this dietary intervention. The selected miRNAs were those differentially expressed in responders and non-responders because, considering that a relationship was found between an altered miRNA profile and an impaired glycaemic control in this cohort, the hypothesis was that grape pomace supplementation could revert, at least partially, the altered miRNA profile observed in the responder subjects. Comparisons between responders and non-responders at the end of the GP supplementation were not assessed because, due to the fact that significant differences at the basal stage were confirmed between both groups [21], the statistical analysis would necessarily include not only the differences at the end of the GP supplementation, but also the differences at the basal stage and, consequently, an important bias would be introduced.

When comparing the expression of the selected miRNAs before and after grape pomace supplementation in the whole cohort, among the seven miRNAs analysed, significant changes in miR-23a, miR-30c, and miR-181a expressions, but not in that of miR-let-7a and miR-10a, were observed, in addition to a change in miR-222 and miR-151a. Consistent with the search for target genes in the MiRecords database, no validated target genes were described for these miRNAs. As predicted, all of these miRNAs had target genes involved in glycaemic control, with the exception of miR-151a (Table 1). By analysing the expression of these miRNAs in responders and non-responders individually, it was found that the increase observed in miR-23a in the whole cohort was present in both subgroups of participants. The increase in miR-181a was significant among non-responders (76%) but not responders (46%). The decrease in miR-30c and miR-222 was found in the responders, but not in the non-responders. The microRNA miR-23a was discarded as a mediator of the beneficial effects of grape pomace on the grounds that the effect of this dietary supplementation on their expression was the same in both responders and non-responders. Moreover, miR-10a was rejected by virtue of a lack of changes in the entire cohort. Finally, miR-151a, miR-181a, and miR-let-7a were discarded because the changes induced by grape pomace were only observed in non-responders. Based on these results, this discussion focuses on miR-30a and miR-222.

Regarding miR-30c, increased expression has been reported in mice fed with a high-fat diet, which developed insulin resistance and obesity, two metabolic syndrome risk factors. In humans, miR-30c was up-regulated in ectosome-enriched plasma of patients who suffered from type 2 diabetes [31,32]. Moreover, in women with gestational diabetes mellitus, miR-30c was significantly increased in cord blood compared with the controls [33]. These results suggest that miR-30c is negatively related to glycaemic control because, in situations in which glucose homeostasis is impaired, this miRNA increases. In our previous study, which employed the present cohort, it was established that responder subjects were those who showed a more altered glycaemic control. These subjects displayed a higher expression of miR-30c than non-responders. Consequently, this data is in good accordance with the reported literature [31,32]. The supplementation with grape pomace improved glycaemic control in these subjects and this effect was paralleled by a significant decrease in the expression of miR-30c, a change that was not observed in the subjects who did not positively respond to this supplementation. These results support the negative role of miR-30c in glycaemic control, and show the potential role of this miRNA in the anti-diabetic effects of grape pomace. To gain additional insight into this issue, a search for the predicted target genes of this miRNA was performed: PKM (pyruvate kinase), TKT (transketolase), PIK3R2 (phosphoinositide-3-kinase regulatory subunit 2), PRKAB2 (protein kinase AMP-activated non-catalytic subunit beta 2), and SOCS1 and SOCS3 (suppressor of cytokine signalling 1 and 3).

**Figure 4:**
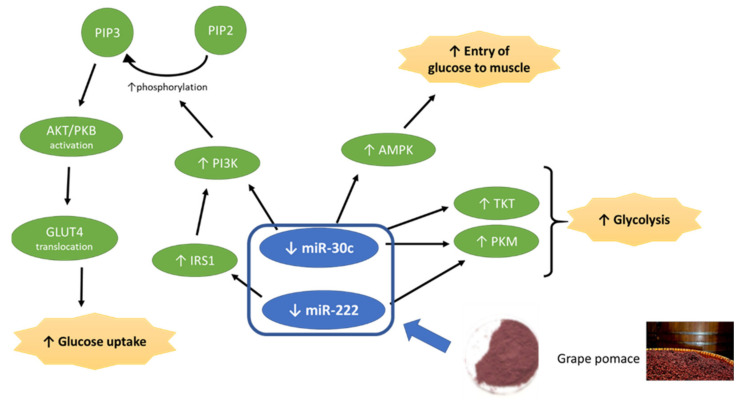
Effects of grape pomace on miR-30c and miR-222 and potential consequences in metabolic pathways involved in glycaemic control.

Pyruvate kinase catalyses the final step of glycolysis. Several authors have described that, in subjects with disturbed glucose metabolism, glycolysis is significantly impaired compared with subjects showing normal glucose metabolism. In specific cases of type 2 diabetes, a decreased glycolysis rate has been observed [34,35]. Moreover, it has been reported that transketolase deficiency decreases glycolysis [35]. Based on these facts, and considering that these two enzymes are miR-30c targets, it can be proposed that the improvement in insulin action induced by grape pomace in responder subjects may be mediated by the aforementioned miRNA. Thus, the reduction in its expression induced by grape pomace supplementation would lead to an increased expression of these proteins and consequently to increased glycolysis (Figure 4).

Another target of miR-30c is the regulatory subunit of phosphoinositide-3-kinase (PI3K), which phosphorylates phosphatidylinositol 4,5-bisphosphate to generate phosphatidylinositol 3,4,5-trisphosphate (PIP3) which, in turn, allows the recruitment of AKT1 to the membrane [36]. This enzyme plays a key role in glucose tolerance improvement by facilitating the effect of insulin. Protein kinase AMP-activated non-catalytic subunit beta 2 is a regulatory subunit of the AMP-activated protein kinase (AMPK), an enzyme which enables the entry of glucose into the muscle in an insulin-independent manner [37]. In view of these facts, it can be proposed that, by means of the decrease in miR-30c, grape pomace may increment the expression of these two proteins, leading to increased glucose uptake though both insulin dependent and independent pathways (Figure 4).

With regard to mRNA expression of SOCS 1 and SOCS3, almost all insulin-sensitive tissues showed a strong up-regulation in rodent models of insulin resistance, revealing a robust and direct relationship between insulin resistance and SOCS [38,39,40]. SOCS3 over-expression decreases the levels of IRS1 tyrosine phosphorylation and inhibits the phosphatidylinositol-3 kinase (PI3K) activity, a downstream signalling element of IRS1 [40,41]. Thus, SOCS3 is critical for the inhibition of the insulin-signalling pathway. These results suggest that the suppression of SOCS3 is beneficial for the activation of insulin signalling.

It has been established in the present study that the reduction in mir-30c may lead to an increase in SOCS3, an effect that can be considered to be negative. In spite of this potential effect, glycaemic control was improved in responder subjects. Based on this assumption, two hypotheses can be proposed. First, the potential activation of IRS-1 via miR-222 may be higher than the probable inhibition induced by SOCS3 via miR-30a. Second, it has been reported that resveratrol, a polyphenol present in grapes, reduces SOCS3 protein expression [42,43]. Hence, it can be hypothesised that this effect may counteract the potential increment induced by decreased miR-30c expression in responders.

The relationship between miR-222 and glycaemic control has been well documented in the literature. Its over-expression can produce glucose intolerance in animal models, due to impaired insulin production and secretion from pancreatic β-cells [44]. In a study carried out by Li et al. (2020), it was stated that miR-222 expression was significantly elevated in serum, serum exosomes, and gonadal white adipose tissue of mice fed with a high-fat diet. Accordingly, there was a corresponding down-regulation of IRS1 and phospho-AKT levels in their liver and skeletal muscle tissues, which correlated with impaired insulin sensitivity and glucose intolerance [45]. In a similar manner, miR-222 has been shown to be up-regulated in type 2 diabetic subjects and in pre-diabetic subjects [46,47]. In 2017, Vivacqua et al. published a review in which the up-regulation of miR-122 in insulin resistance was highlighted [48]. In the present study, the expression of this miRNA was reduced in responder subjects after supplementation with grape pomace, which is rich in proanthocyanidins. This result is in good accordance with that reported by Castell-Auví et al. (2013), who showed the down-regulation of miR-222 in rats treated for 45 days with 25 mg of procyanidins per kg of body weight per day [49]. According to the search in the MiRecords database, miR-222 and miR-30c share PKM as a potential target gene and, thus, it may play a role in a possible increase in glycolysis induced by grape pomace (Figure 3).

As described in the Material and Methods section, in addition to the search in the MiRecords database, a search in the literature for other target genes was performed. As a result, it was found that Ono et al. demonstrated that both mouse and human IRS-1 mRNAs are direct targets of miR-222 [26]. Based on this result, an increase in IRS-1 protein expression leading to improved insulin action may be proposed as one of the mechanisms of action to explain the beneficial effect of grape pomace.

The present study was subject to some limitations. The size of the sample group was relatively small, which calls for further validation in larger samples. Moreover, the actual effects of the changes observed in miRNAs expression on genes could not be experimentally analysed on the grounds that no tissue biopsies for this cohort were available, and only plasma samples were used, which were more easily accessible. For the same reason, miRNAs were measured in plasma rather than in tissues. Nevertheless, a positive correlation between miRNAs in plasma and other biological fluids, such as urine and bile, and tissue miRNAs has been demonstrated in healthy humans [50].

## 5. Conclusions

The present study shows that miR-30c and miR-222, two microRNAs associated with insulin resistance and diabetes, may have a pivotal role in the improvement of glycaemic control induced by grape pomace supplementation. Consequently, these miRNAs can be suggested as potential biomarkers of the efficacy of this dietary approach. Nevertheless, considering that the mechanistic conclusions are mainly based on bioinformatics, further functional studies are warranted to increase the scientific evidence supporting them.

## Figures and Tables

**Table 1:** Predicted target genes according to the MiRecords database and validated target genes found in the literature for the microRNAs analysed in the present study, which are related to glycaemic control.

miRNA	Predicted Target Genes MiRecords Data Base	Validated Target Genes in the Literature
hsa-mir-222	PKM	IRS-1 [26]
hsa-let-7a	IGF2BP1, IGF2BP3 ADCY9, IRS2	
hsa-mir-10a	-	GLUT1 [27]
hsa-mir-151a	-	
hsa-mir-181a-5p	IRS2, SIRT1, ACOT12	
hsa-mir-23a-3p	PKM, PPARGC1A	
hsa-mir-30c-5p	PKM, TKT; PIK3R2, PRKAB2, SOCS1; SOCS3	

PKM: pyruvate kinase, IRS-1 and IRS-2: insulin receptor substrate 1 and 2, IGF2BP1 and IGF2BP3: Insulin-like growth factor 2 mRNA-binding protein 1 and 3, ACDY9: adenylate cyclase 9, GLUT1: glucose transporter 1SIRT1: NAD-dependent deacetylase sirtuin-1, ACOT12: Acyl-CoA thioesterase 12, PKM: pyruvate kinase, PPARGC1A: peroxisome proliferator-activated receptor gamma coactivator 1-alpha, TKT: transketolase, PIK3R2: phosphoinositide-3-kinase regulatory subunit 2, PRKAB2: protein kinase AMP-activated non-catalytic subunit beta 2, SOCS1 and SOCS3: suppressor of cytokine signalling 1 and 3.

## Data Availability

The data presented in this study are available on request from the corresponding author. The data are not publicly available.

## Supplementary Materials

The following are available online at https://www.mdpi.com/article/10.3390/foods10092059/s1, Table S1: Targeted miRNA sequences (source miRbase.org).
