# Supplementary material for: Potential Relationship between the Changes in Circulating microRNAs and the Improvement in Glycaemic Control Induced by Grape Pomace Supplementation
Source: Foods. 2021 Sep 1;10(9):2059. doi: 10.3390/foods10092059 (PMC8470177; doi:10.3390/foods10092059)
Supplement: Supplementary file 1 [file foods-10-02059-s001.zip › foods-1302232 supplementary matterials.pdf]

Table S1. Targeted miRNA sequences (source miRbase.org).

hsa-miR-30c-1-5p: 3′-UGUAAACAUCCUACACUCUCAGC-5′
hsa-miR-23a-3p: 3′-AUCACAUUGCCAGGGAUUUCC-5′
hsa-miR-222-3p: 3′-AGCUACAUCUGGCUACUGGGU-5′
hsa-miR-let7a-1: 3′-UGAGGUAGUAGGUUGUAUAGUU-5′
hsa-miR-151a-5p: 3′-UCGAGGAGCUCACAGUCUAGU-5′
hsa-miR-181a-1-5p: 3′-AACAUUCAACGCUGUCGGUGAGU-5′
hsa-miR-10a-5p: 3′-UACCCUGUAGAUCCGAAUUUGUG-5′
